# A Treatise on Diseases and Injuries of the Nerves

**Published:** 1834-04-01

**Authors:** 


*A Treatise on Diseases and Injuries of the Nerves.* By Joseph Swan. *A new Edition, very considerably enlarged.*—London, 1834. 8vo. pp. 356, and ten plates.

In our last number we had the pleasure of giving some account of Mr. Swan's method of demonstrating the nerves of the dead; we have now the greater satisfaction of analysing his treatise on those morbid affections whose seat is in the nerves of the living. In this, as in the former work, Mr. Swan, to use a familiar expression, is quite at home, and every page shews the mastery of a great subject which may be attained by an acute mind studying it through a long series of years, with an attention proportioned to its importance. Our author's style is that of a man who can well afford to despise ornament; it is characterised by an antique simplicity, the internal evidence of the accuracy of the facts narrated. This excellent treatise is indispensable to every one who wishes to acquire a sound knowledge of the subject to which it relates; nor will the future historian of the diseases of the nerves, however brilliant his talents, however vast his acquirements, be justified in passing over the researches of Mr. Swan: it will be long indeed before they are obsolete.

The first chapter treats "of painful affections of nerves in general," and commences in the following manner:

"The nerves most subjected to painful affections are the fifth, and those arising from the whole length of the spinal chord. It may be expected that I should state whether pain be felt only by the posterior bundles of the spinal nerves, which have been termed sensitive, and not by the anterior, or those termed muscular. When a muscular nerve is irritated, an immediate contraction of the corresponding muscle takes place; it must therefore be endowed with perceptibility; but whether this be cognizable by the mind as a sensation in the common acceptation of this quality, is very difficult to determine; nor is it less so whether its morbid condition be productive of pain. The facial portion of the seventh pair of nerves conveys the power of action to many of the muscles of the face, but is subjected to painful sensations. Experiments on the par vagum have given rise to various opinions. When this nerve has been either divided or inclosed in a ligature, the animals have expressed the most uncomfortable sensations, but whether these have been feelings of ordinary pain, or only suffocation, I cannot determine; but it may have been either one or the other, as its disorder produces a sense of suffocation in the lungs and pain in the stomach. The morbid condition of a single nerve, and particularly of part only of its origin, can seldom come under the consideration of the pathologist; for, almost as soon as it has left the brain or spinal chord, it has either communicated or become intermixed with other nerves. When it has been affected at its seat in the brain, some other portion of this organ, besides the precise spot giving origin to it, has been implicated in the disease. Experiments for determining the sentient qualities of nerves have not been so satisfactory as to have carried conviction to the minds of unbiassed persons; for, when animals of the same species have been subjected to the same treatment, one has expressed the keenest suffering, whilst another has been altogether unmoved. But observe what takes place on the performance of an experiment. Incisions are made through sensitive parts: these are forcibly retracted for allowing the separation of the specific nerve from its surrounding connexions, and the state of the wound is momentarily varying, so that, when the nerve itself is irritated, it is impossible in many instances for the most candid observers to form a correct judgment." (P. 1.)

It is difficult, we think, to determine whether it is really the facial portion of the seventh pair of nerves which suffers pain, as it always has filaments of the fifth pair distributed with it, and running in the same sheath. The observations in the text on the doubtful results of experiments are extremely just, and shew especially how little value can be attached to negative evidence in cases of this kind; but it is obvious that even the affirmative results may be obscured by "the state of the wound," and other circumstances.

Our author observes shortly afterwards, "In one of the cases of a wounded thumb, related in the chapter on partial division of nerves, motions could be observed in different parts; and, from the description of the patient, I cannot help thinking that what she called spasms were contractions of the nerves producing shocks. These motions are not always attended with pain; but I conceive that contractions of the nerves may take place and produce pain, in the same manner as those of the muscles during their violent action in cramp or tetanus." (P. 5.) We should rather suppose these motions, as well as a quivering of the cutaneous nerves underneath the skin or the outside of the leg, of which our author speaks, to depend on an undulation of the nerves; or, if the nerves circulate a fluid, as some have plausibly conjectured, these phenomena might arise from their distension.

The following observations on intermittent neuralgia are judicious and instructive:

"Pain affecting the nerves comes on in momentary shocks at irregular periods, but in some cases begins at one particular hour, and is continued for a longer or shorter period. When it comes on regularly, it is presumed, from its similitude to the return of ague, that it is not an affection of the nerves, but this is a mistaken notion; it is, however, a curious circumstance, and worthy of due consideration. The following case shows that it may be entirely owing to the irritation of a nerve:

"CASE. Charles Chelsop, aged thirty years, was admitted into the Lincoln County Hospital, on the 14th of August, 1820, with an aneurism in the right ham. He dated its commencement from a fall he had from his horse about twelve months before. The aneurism had been very painful for the five preceding weeks, and at the time of his admission the whole leg was œdematous, and in a state of inflammation; but these symptoms were considerably diminished by confinement to bed, and taking some purging medicine.

"On the 19th I tied the femoral artery with a single ligature of strong silk. The ends of the ligature were left hanging out of the wound. The greatest part of the wound united by the first intention. There was no disorder of the constitution. The ligature came away on the 5th of September, when, as the wound appeared healed, he walked out of doors, and was discharged from the hospital quite well on the 9th.

"For five weeks before the operation, he told me, the violent pain came on in the leg every night about half-past ten, and went off about two in the morning. It appeared to be entirely in the sciatic nerve. This might have been accounted for by supposing him to have lain so as to press the nerve in the upper part of the thigh, as it always came on in the night; but it returned just at the same time for several days before the operation, when he was entirely confined to his bed. It was never felt after the operation.

"In the same manner I have known an ulcer placed over cutaneous nerves near the ankle to be affected by the most excruciating pain, at the same hour early every morning, whilst other ulcers on the same leg were hardly noticed. The subject of this case died of apoplexy.

"All local affections of nerves appear to come on, or at least to be aggravated, periodically. Why this should be so it is difficult to explain. It may be that a nerve cannot at first bear a diseased action without rest, any more than it can a healthy one, and therefore the diseased action, after a certain period, ceases to make any impression. But, after this rest, the nerve acquires fresh power, and is again fitted for the same action.

"In the case of Charles Chelsop, the structure of the nerve could not have been altered from the pressure of the aneurism; but I conceive the aneurism irritated it, and, at the particular time of the approach of violent pain, produced some change in the nerve. It may be supposed that the aneurism itself became enlarged at this period, either from a greater determination of arterial blood to it, or some impediment to the free return of this by the veins, and thus extended the nerve to a greater degree, or that there was an increased action of the blood-vessels of the nerve itself, or that the nerve was in a state of spasm or contraction, and during the period of ease became passive and yielding from exhaustion, and incapable of being acted upon without further rest." (P. 12.)

In the second chapter, which treats "of painful affections in particular nerves," we find two interesting cases of neuralgia cured by bark.

"CASE. Mr. M., seventeen years of age, applied to me on account of an excruciating pain in his face, for which he had used remedies prescribed for him without any relief. About two months before, he received a blow on his face, which broke one of the middle incisive teeth of the upper jaw; some swelling of the face was occasioned by the injury, but in a short time it entirely subsided. When I saw him there did not appear to be any particular cause for the pain, as his general health was not much impaired, although he had become thinner by the continuance of the pain. I ordered him to take two scruples of powdered bark with the following draught every three hours, and to drink several glasses of wine daily:—℞. Decoct. Cinchon., Infus. Caryoph. *aa.* ℥vj.; Syrupi ℥j. M. Ft. Haust.

"After the use of these remedies for a short time the pain entirely left him, but confinement again brought it on, when recourse being had to the bark in larger doses, the pain gradually subsided, and he afterwards had no relapse.

"I have before observed, that whatever tends to weaken the body produces a morbid sensibility of the nervous system, and disposes to the production of disordered functions of the nerves. In the following case the complaint came on in consequence of poison taken into the stomach.

"CASE. Mr. B., having eaten some hashed hare, became ill, with others who partook of it. It was afterwards discovered to have stood in a brass pan, which was found on examination to be covered with verdigris. From this time he was never well; and when I saw him, some months after, he had an affection of the nerves at the back part of the head, which caused excruciating pain, and had tormented him a long time. He had become very weak and much emaciated in consequence of the almost continual severity of the pain, and, in short, he quite despaired of ever being better. He had used a variety of remedies, without any abatement of the complaint.

"I ordered him half a drachm of powdered bark, to be taken every three hours, and a blister to be applied to the back of the neck; and, as the pain was usually more severe in the morning, to have a draught, with thirty drops of laudanum, to be taken at that time; and I recommended him to drink wine and malt liquor. Soon after he was put on this plan of treatment the pain began to be less severe, and it kept gradually diminishing, and at length quite left him. As the pain diminished, his bodily strength increased; and in the course of some months he became as strong as he had ever been at any period of his life. At first the bark was taken regularly every three or four hours, but afterwards not quite so regularly, and it was continued altogether for about six weeks." (P. 40.)

When treating of partially divided nerves, Mr. Swan has the following practical remarks:

"I have very little doubt but by far the greatest number of injured nerves in venesection is made troublesome by using the arm too soon, and bringing on inflammation; for I have never seen any bad consequences in those patients who have been so ill as to be unable to do anything.

"Whenever a person has complained of very acute pain on the opening of a vein, great care ought to be taken to close the wound well with a linen compress and bandage, and to keep these continually on the orifice, and at the same time to confine the arm in a sling until the wound is perfectly healed.

"I relate the following case, to show that if a nerve be injured in bleeding, and the external wound heal by the first intention, that of the nerve may not be of any consequence.

"Case. I bled Mrs. D. in the median cephalic vein; she complained of very acute pain at the time I made the puncture, and it continued for several hours.

"As I was certain from the manner in which she complained that I had wounded a nerve, I was very careful in binding up the arm, so as to keep the lips of the wound in exact contact, and at the same time told her of the necessity there was for keeping her arm entirely at rest. The wound healed by the first intention, and the pain did not return.

"Some have supposed that the injury of a nerve in venesection is always a partial division, and that in order to a cure, it is necessary to be wholly divided. That it is so sometimes, I shall relate cases to prove; but that it is not always so, will appear from the following.

"Case. A woman about forty years of age got a fall, which shook her head very much, and for which I bled her from the cephalic vein; she felt no inconvenience for two or three days, but after that her arm became painful from the shoulder to the wrist, and she seemed to suffer very much.

"There was a little thickening about the cicatrix, and it was painful when touched. I ordered some extract of belladonna to be applied to it. Some days after, when I saw her, the cicatrix was quite even and soft, and might be pinched without producing pain. The pain now only struck upwards to the neck whenever she attempted to straighten her arm. I ordered her arm and neck to be bathed with the following liniment, and the pain gradually wore off, although it was some weeks in doing so:—℞. Linim. Carb. Amm. ʒvj., Tinct. Opii. ʒij. M. ft. Linim.' (P. 115.)

"When the ears are stopped," says our author, "and a watch is brought in contact with any part of the head, face, teeth, or neck; or if a stick, water, &c. be interposed between any of these parts and the watch, the sound will be heard as well as when the ears are open." (P. 279.) He does not side with those who suppose that this depends merely on the mechanical conveyance of the sound, as the sound would then be heard, at whatever part of the head or face the watch were applied, which is not the case; but he thinks, with great reason, that the conveyance of the sound depends on a communication between the auditory and facial nerves at the bottom of the internal auditory meatus. The bearing of this interesting fact, first discovered by our author, will be obvious from the following case, with which we must reluctantly close our extracts.

"Elizabeth Nobles, aged thirty-six years, was born with the external auditory meatus of each ear imperforate. In the right auricle there is a very slight trace of the external auditory meatus, and there appears to be no other part of the auricle but part of the helix and the lobe. In the left there is a slight trace of the meatus, but it is only about one sixteenth of an inch deep; there is the form of the auricle, but the different eminences are not distinct, and the communication between the external air and the membrane of the tympanum of each ear, if these exist, is as completely obstructed as is possible. She did not begin to talk at all intelligibly till she was seven years old, and she did not talk tolerably well before she was about twelve: she can now talk to be perfectly understood. She can hear perfectly when a person addresses her at the distance of six or seven yards. She cannot hear so well, when the person speaking is behind her. She cannot hear a watch unless it is in contact with her face, and not if it is in her mouth, unless it is in contact with some part of this. She herself, as well as others who have known her, supposes that she hears through her mouth and nose, and from observing the motions of the lips. To prove that this is not the case, the circumstance I have related with respect to the watch might suffice, but I made her shut her eyes, and she heard distinctly what I said, as likewise when both her mouth and nose were quite closed. Putting a cloth over her mouth, and nipping her nose, have made a slight difference now and then; but nothing more than I could suppose would happen from the extent of the face the sound was thus kept from. I have pressed my fingers on the parts where the external auditory meatus should be, but she heard just as well as before. I have put a thick cloth over them, and at the same time pressed this as close as I could, but it made no difference. I stood four feet from her after having put a linen cloth over her face, and when I addressed her, she heard distinctly. I then put over the linen a piece of flannel, and she still heard me. I then put over the flannel a large woollen cloth coat, and asked her several questions, but she could not hear any of them. I removed all the coverings, and used the same words in the same tone, which she told me immediately. I made the same experiment another day, but she heard all the questions I asked, though more faintly, according to the covering put on her face. Some variation will always exist in these experiments, for it is impossible always to remember the exact tone of voice made use of in them, and some little difference may likewise exist in the coverings. She could hear distinctly tunes played on the piano-forte at the distance of seven or eight feet, and I covered her face, as in the other experiments, and the sounds were fainter. I pressed on her ears with a cloth, but she heard the tunes just as well. I placed her in a chair near the piano, and covered her face so as to hinder her from hearing so well; I then placed her hand on the piano, and she heard much better: I then tied a silk hand-kerchief tight round the arm, and she did not hear so well, and she heard better again when I loosened it. She cannot hear the sound of bells at a distance; nor hear the cathedral clock strike unless she is very near, though the hammer strikes on a bell, which is one of the largest in the kingdom, and can be heard at different times at the distance of several miles.

" When her face or teeth are in contact with the piano, on which any one is playing, the sound is very loud to her." (P. 283.)

The knowledge of this fact will be of inestimable value in the education of the deaf and dumb; as in many cases these unfortunate beings do not suffer from any defect of the auditory nerve.

The plates with which this treatise is illustrated do great credit to the artists whom Mr. Swan has employed: two of them are of extraordinary beauty; one representing the sciatic nerve, with some of its fibrils lacerated, and surrounded by a coagulum of blood; the other shewing a partial division of one of the digital nerves.

It is needless to repeat our opinion of this admirable treatise. Its price is very moderate; the book ought to be in everybody's hands, and the substance in everybody's head.

